# Polymer Concentration-Driven Morphological and Mechanical Variations in Flash-Spun High-Density Polyethylene Fibers

**DOI:** 10.3390/polym17070965

**Published:** 2025-04-01

**Authors:** Jae-Hyung Wee, Younghwan Bae, Nam Pil Cho, Minsung Kang, In-Woo Nam, Hyunchul Ahn, Donghwa Ryu, Seung Goo Lee, Tae Hee Han, Sang Young Yeo

**Affiliations:** 1 Textile Innovation R&D Department, Korea Institute of Industrial Technology, Ansan-si 15588, Republic of Korea; jhwee0506@kitech.re.kr (J.-H.W.); yhwanee@kitech.re.kr (Y.B.); onefalse@kitech.re.kr (M.K.);; 2 Department of Applied Organic Materials Engineering, Chungnam National University, Daejeon 34134, Republic of Korea; 3 Department of Organic and Nano Engineering, Hanyang University, Seoul 04763, Republic of Korea; 4 Department of Fiber System Engineering, Yeungnam University, Gyeongsan 38541, Republic of Korea; hyunchul.ahn@yu.ac.kr; 5 Department of Carbon Convergence Engineering, Jeonju University, Jeonju 55069, Republic of Korea; yjj444@hanmail.net

**Keywords:** flash-spun filaments, polymer concentration, high-density polyethylene, mechanical properties, crystallization behavior

## Abstract

Flash-spun filaments (FSFs) made from high-density polyethylene (HDPE) are widely used in industrial nonwovens due to their unique morphology and mechanical robustness. In this study, we investigated the effect of polymer concentration (5–15 wt%) on FSF formation using a laboratory-scale flash-spinning system operating under supercritical conditions. Morphological, mechanical, and crystallographic analyses were conducted to understand the underlying mechanisms. As polymer concentration increased, filament thickness, crystallinity, and strength improved, with optimal performance observed at 12 wt%, where the modulus peaked at 270.77 cN/tex and elongation was minimized. At 15 wt%, mechanical properties declined due to hindered solvent evaporation, which disrupted polymer alignment and reduced filament orientation. X-ray diffraction analysis revealed small crystal sizes (6.4–6.9 nm) across all samples, suggesting that rapid phase separation limited crystal growth. This indicates that polymer concentration mainly affects the number of crystalline domains rather than their size. The results demonstrate that solvent evaporation dynamics and phase separation behavior play critical roles in determining FSF structure and performance. Precise control of polymer concentration is therefore essential to optimize fiber morphology, orientation, and mechanical stability, providing valuable insights for the design of high-performance flash-spun nonwovens in industrial applications.

## 1. Introduction

Industrial nonwovens play a vital role in modern society owing to their versatility and functionality. These materials are indispensable in various sectors, such as healthcare, construction, automotive, and filtration, which require properties such as lightweight structures, durability, and cost-efficiency [1,2,3,4]. In healthcare, nonwoven materials are widely used in surgical masks and gowns, offering critical protection while remaining breathable and disposable. In the construction industry, they serve as geotextiles and roofing underlays, providing strength and stability in demanding environments. The increasing demand for advanced materials across these sectors has driven the development of innovative manufacturing processes for producing nonwovens with enhanced performance characteristics.

Flash-spun nonwovens (FSNWs) represent a significant advancement in the nonwoven technology [4]. These nonwovens are created by a process that results in unique fibrillation, producing ultrafine fibers with net-like structures [5,6], which are ideal for high-performance applications such as protective clothing, waterproof breathable membranes, and building materials; for example, Tyvek^®^ is a well-known FSNW product for protective garments and weather-resistant house wraps. The performance and durability of FSNWs are influenced by their mechanical properties, including tensile strength, tear resistance, and flexibility; for example, the material can withstand rigorous use in protective clothing owing to its high tensile strength, whereas its tear resistance is essential for applications such as construction membranes that are subjected to external stresses [7].

Flash spinning is an advanced technique for producing microfibers, particularly for high-performance industrial nonwovens [8,9]. This process involves dissolving a molten polymer, such as olefin polymers (e.g., polyethylene and polypropylene), in a supercritical phase solvent, such as halocarbon-based compounds (e.g., trichlorofluoromethane (CFC-11) and dichlorotrifluoromethylmethane (HCFC-123)) or hydrocarbons such as pentane and hexane [10,11,12]. High-density polyethylene (HDPE) is especially favored in flash spinning owing to its semicrystalline structure, which facilitates the formation of strong, interconnected fibrils that are critical to the unique network morphology of FSNWs [13]. Additionally, HDPE exhibits properties such as high tensile strength, chemical resistance, durability, and thermal stability and is thus ideal for demanding applications, including protective garments, filtration membranes, and packaging materials. Its low density and compatibility with supercritical solvents enable efficient dissolution and phase separation during the process, while its thermal stability allows it to maintain structural integrity during high-temperature, high-pressure operations [14,15,16].

During flash spinning, solvents act as supercritical fluids, exhibiting liquid-like densities and gas-like diffusivity above their critical temperature and pressure [17]. This unique state enables the supercritical fluid to disrupt polymer–polymer interactions and penetrate crystalline regions, dissolving semicrystalline polymers such as HDPE effectively [11,18]. The polymer–solvent solution is then ejected to ambient conditions through a nozzle, and this abrupt pressure drop induces simultaneous phase separation and solvent evaporation [19], resulting in the formation of solid filaments, which are subsequently processed into FSNWs. Compared to conventional techniques such as melt spinning or solution spinning [20,21], flash spinning offers the distinct advantage of creating highly interconnected fiber networks in a single step, resulting in materials with exceptional strength, barrier properties, and uniformity. In melt spinning, fibers are formed by extruding molten polymer through a spinneret and cooling it to solidify, which limits the achievable microstructure and network complexity. In solution spinning, the polymer is dissolved in a solvent and solidified by coagulation or evaporation, typically requiring additional processing steps and producing less entangled morphologies. In contrast, flash spinning combines rapid phase separation and explosive solvent evaporation, generating a three-dimensional, fibrillated network structure without the need for post-processing or external coagulation, making it especially suitable for high-barrier nonwoven applications.

Understanding the dynamics of the filament formation process is crucial to optimize the performance of FSNWs. Key parameters of the flash-spinning process—pressure, temperature, and polymer concentration—alter the rheological behavior of the polymer solution, thus significantly influencing the quality of the produced fibers [12,22,23]. Pressure, particularly the phase separation pressure (PSP), is crucial for determining the morphology, strand-diameter distribution, and crystallinity of flash-spun filaments (FSFs) [22]. Studies have shown that an optimal PSP of 76 bar at an HDPE concentration of 8 wt% results in fibers with high crystallinity and tensile strength, whereas an excessively high or low PSP affects mechanical performance. Polymer concentration is equally important, as it directly impacts the solution viscosity, affecting filament morphology and mechanical properties [12,24]. Despite the recognized importance of these individual parameters, the interplay between pressure and polymer concentration remains relatively underexplored, presenting opportunities for further optimization of the process. Further studies on the interplay among polymer concentration, solvent properties, and process conditions are necessary to fully understand the flash spinning process and optimize filament formation. Such insights can aid in producing high-performance nonwovens with tailored mechanical and barrier properties for a wide range of industrial applications.

In this study, we investigated the influence of polymer concentration on the properties of flash-spun high-density polyethylene (HDPE) filaments using a lab-scale supercritical system. Unlike conventional studies focused on melt or gel spinning, our work systematically analyzes how concentration affects morphology, mechanical strength, and crystallization in flash-spun fibers. By correlating solvent evaporation, polymer alignment, and fiber performance, we identify 12 wt% as a critical threshold for optimized filament integrity. These findings provide valuable insights for improving process control and advancing industrial applications of flash-spun nonwovens.

## 2. Materials and Methods

### 2.1. Materials

HDPE (E308 grade) was purchased from KOREA PETROCHEMICAL Co. (Seoul, Republic of Korea), with a melting temperature (T_m_) of 134 °C, a melt index of 0.85 at 190 °C, and a density of 0.956 g/cm^3^. CFC-11 solvent (boiling point: 23.8 °C; critical temperature: 197.9 °C; critical pressure: 43.9 bar; density: 1.48 g/cm^3^) was obtained from NOW ENG Co. (Asan, Republic of Korea) and filtered twice using a microfilter before use.

### 2.2. Flash-Spinning Process of HDPE

Flash spinning was performed using a laboratory-scale system comprising a stainless-steel high-pressure vessel (500 mL capacity, rated up to 350 bar), a multistage spinning nozzle assembly, and a high-pressure N_2_ accumulator. The nozzle system, with 0.7-mm-diameter primary and secondary nozzles, included a 4.71-mL phase-separation space to facilitate pressure reduction. HDPE was dissolved in the solvent with concentrations of 5, 8, 10, 12, and 15 wt%, occupying 82% of the vessel’s volume. The vessel was heated to 220 °C at 5 °C/min while being stirred at 300 rpm. When the critical pressure was exceeded, the polymer solution was ejected through the nozzle. The pressure drop was controlled using a nitrogen accumulator maintained at a pressure of 200 bar. The filaments produced were named FSF-5, FSF-8, FSF-10, FSF-12, and FSF-15 based on the polymer concentrations (5, 8, 10, 12, and 15 wt%, respectively).

### 2.3. Characterizations

The morphology of the FSFs was observed using scanning electron microscopy (SEM; SU8600, HITACHI, Hitachi, IBR, Japan), and the strand-diameter distribution was analyzed using the ImageJ software (Ver. 1.53e). The thermal properties were examined using differential scanning calorimetry (DSC; TA Instruments, New Castle, DE, USA) at a heating rate of 20 °C/min. Crystallinity was determined from the crystallization enthalpy (ΔH_c_) by integrating the DSC melting curve, using a reference enthalpy of 288 J/g for 100% crystallized HDPE [25]. Microstructural changes were determined by wide-angle X-ray diffraction (WAXD; Xenocs, Grenoble, France) with Cu-Kα radiation, and azimuthal scans at 22° were conducted to confirm the preferred polymer orientation. The mechanical properties of the filaments were evaluated using a universal tensile testing machine (INSTRON, Norwood, MA, USA). The gauge length was set to 100 mm, and the crosshead speed was maintained at 120 mm/min. Mechanical property data (tenacity, tensile elongation, and modulus) were collected from 30 specimens for each polymer concentration to ensure statistical reliability.

## 3. Results and Discussion

**Figure 1:**
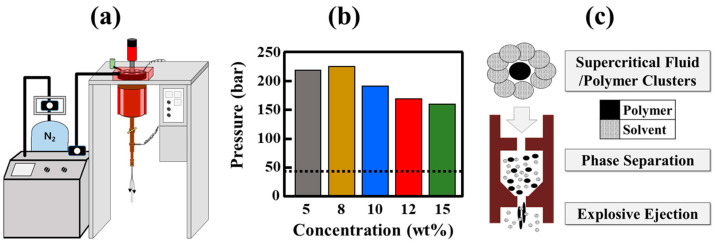
(**a**) Laboratory-scale flash-spinning equipment; (**b**) internal pressure of the high-pressure vessel at 220 °C; and (**c**) schematic of the flash-spinning process (the dotted line in Figure 1b is the critical pressure of the solvent at 220 °C).

FSFs have been produced using various HDPE concentrations in a laboratory-scale flash-spinning system (Figure 1a). The system is operated at high pressures and temperatures to dissolve the polymer in a supercritical solvent. As the temperature of the system increases to 220 °C, the pressure of the CFC-11 and HDPE mixture spontaneously increases because of solvent evaporation (Figure 1b). At 5 wt% HDPE, the pressure maximizes to 218 bar and gradually decreases to 159 bar at 15 wt% HDPE. This reduction in pressure is observed because the solvent ratio decreases upon HDPE addition, resulting in less vaporization. Despite these changes, the temperature and pressure of the spinning solution remain above the T_m_ of HDPE and the critical point of the solvent, respectively. When the temperature and pressure of the spinning solution exceed the critical point of the solvent, the solvent transitions into the supercritical phase, exhibiting a liquid-like density for effective solubilization of HDPE and gas-like diffusivity to penetrate and mix with the polymer matrix. This supercritical behavior ensures homogeneity in the polymer–solvent mixture and facilitates the subsequent phase separation required during flash spinning [17,18]. Phase separation is induced by pressure-induced phase separation (PIPS) in the pressure-reducing section of the multistage nozzle (Figure 1c), followed by explosive ejection through a secondary nozzle. This rapid depressurization causes the solvent to evaporate instantaneously, leading to the formation of solid filaments.

**Figure 2:**
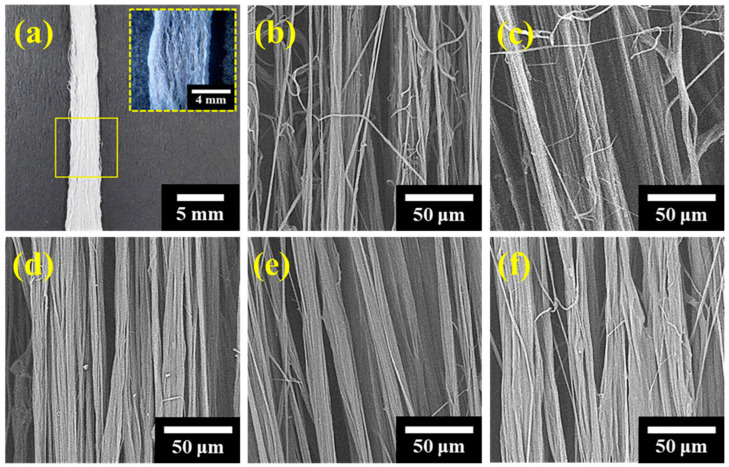
Optical and SEM images of FSFs at different HDPE concentrations: (**a**) Optical image; (**b**) 5 wt%; (**c**) 8 wt%; (**d**) 10 wt%; (**e**) 12 wt%; and (**f**) 15 wt%.

Figure 2 shows the morphologies of the FSFs formed at HDPE concentrations ranging from 5 wt% to 15 wt%. The optical image in Figure 2a highlights the thick, stem-like structure of the FSFs, with the inset showcasing their interconnected network morphology; this structure results from the PIPS during flash spinning [19,26]. The sudden pressure drop reduces the solubility of HDPE, triggering phase separation into polymer- and solvent-rich regions. Simultaneously, the solvent evaporates explosively, elongating and cooling the polymer strands, which solidify to form FSFs with a characteristic three-dimensional interconnected network structure [8,19,27]. The SEM images in Figure 2b–f illustrate the changes in morphology at different HDPE concentrations. At 5 wt%, the network consists of fine, dispersed fibrils with visible gaps. At 8 and 10 wt%, the fibrils become denser and more cohesive, whereas at 12 and 15 wt%, compact networks with uniform fibril diameters are observed. These observations confirm that polymer concentration has a direct effect on the formation of continuous networks. Low concentrations produce only thin strands with a continuous structure, whereas high concentrations form a composite of thick and thin strands.

**Figure 3:**
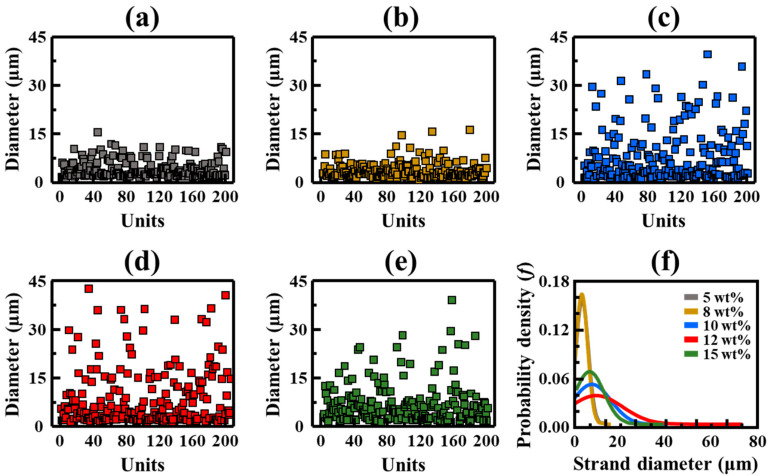
Fineness and strand-diameter distributions of FSFs at different HDPE concentrations: (**a**) 5 wt%; (**b**) 8 wt%; (**c**) 10 wt%; (**d**) 12 wt%; and (**e**) 15 wt%; (**f**) strand-diameter distributions.

Figure 3 and Table 1 indicate the influence of HDPE concentration on the fineness and strand-diameter distribution of the FSFs. Evidently, the linear density (tex), calculated by converting the filament weight to weight per 1000 m [28], increases from 14.1 tex at 5 wt% (FSF-5) to 34.4 tex at 15 wt% (FSF-15; Table 1). This result indicates that high polymer-to-solvent ratios are obtained at high HDPE concentrations, and thus, thick filaments are formed via the expulsion of more polymer during the flash-spinning process. The strand-diameter distribution analysis (Figure 3) further illustrates the structural changes in the FSF networks. The strand diameters are determined from the SEM images (200 strands) using Image J (Ver. 1.53e), and the distribution of the strand diameters is analyzed from the maximum and minimum diameters, standard deviations, and variances using probability density functions. At 5 wt%, FSF-5 exhibits fine strands with an average diameter of 4.17 μm, and most strands show diameters smaller than 15 μm, accompanied by a narrow diameter variance of 7.07, indicating limited polymer chain aggregation. At intermediate concentrations of 8 and 10 wt% (FSF-8 and FSF-10), the average strand diameter drastically increases, with more strands showing diameters greater than 15 μm, reflecting enhanced chain interactions and polymer aggregation due to rapid solvent evaporation. The widest diameter distribution is observed at 12 wt% (FSF-12); at this concentration, the average strand diameter, maximum diameter, and standard deviation increase to 10.17 μm, 73.5 μm, and 11.43, respectively, indicating substantial variability in the formed network structures. At 15 wt% (FSF-15), the strand morphology becomes more uniform, with the average diameter decreasing slightly to 7.42 μm and the diameter variance narrowing, likely due to reduced solvent mobility and limited filament thickening within the dense polymer matrix [12,18,29]. These results highlight the critical role of polymer concentration in controlling FSF morphology. At low polymer concentrations, thin, uniform strands are formed, while at intermediate concentrations, thick and thin strands with increased diameter irregularity are formed together. At higher concentrations, strands with relatively more consistent diameters are produced. These results suggest that during the spinning process of polymer-solvent mixtures, the polymer concentration affects the overall thickness of the FSF, and that evaporation dynamics, such as solvent evaporation and desorption within the phase-separated polymer matrix, significantly affect the strand diameter composition that constitutes the FSF.

**Figure 4:**
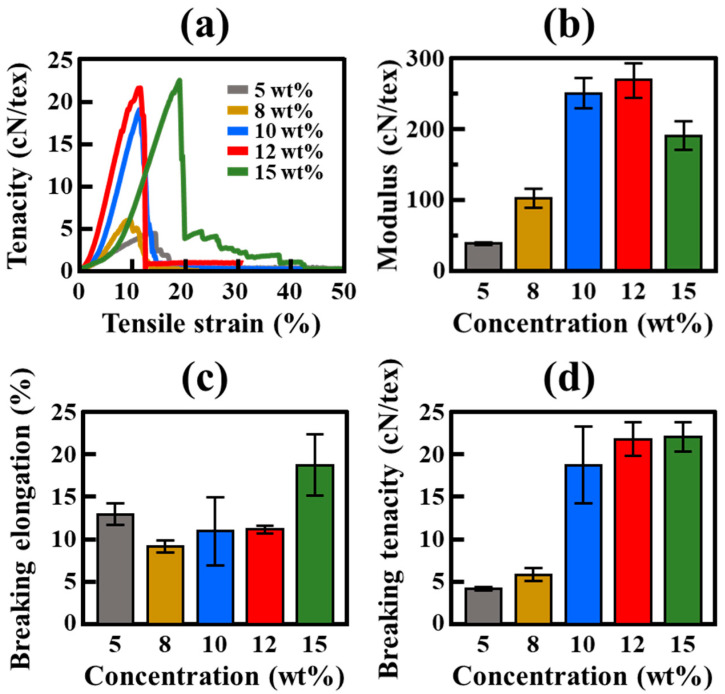
Mechanical properties of FSFs at different HDPE concentrations: (**a**) stress–strain curves; (**b**) modulus; (**c**) elongation; and (**d**) tenacity.

Next, tensile tests were performed to analyze the effect of polymer concentration on the mechanical properties of the FSFs (Figure 4). The tensile strain–tenacity curves in Figure 4a show a progressive increase in tenacity with increasing HDPE concentration. Notably, FSF-5 (5 wt%) exhibits low tenacity and premature failure, indicating weak filament formation due to insufficient polymer aggregation. As the polymer concentration increases (FSF-8 (8 wt%) and FSF-10 (10 wt%)), the FSFs display higher tenacity and improved strain resistance. FSF-12 (12 wt%) achieves the highest tenacity, demonstrating a well-developed filament structure with optimized polymer entanglement and phase separation. FSF-15 (15 wt%) exhibits slightly increased tenacity; however, its strain response improves, indicating a shift toward ductility enhancement at the cost of stiffness.

To further analyze the effect of polymer concentration on the FSFs in more detail, their elastic modulus, breaking strength, and breaking elongation were determined from the tensile strain–tenacity curve (Figure 4a). The modulus of the FSFs, shown in Figure 4b, significantly increases (from 38.81 cN/tex at 5 wt% (FSF-5) to a peak value of 270.77 cN/tex at 12 wt% (FSF-12)) with increasing polymer concentration. This result suggests that moderate polymer concentrations enhance fiber stiffness by improving molecular interactions and filament cohesion [22,23]. However, upon further increasing the concentration to 15 wt% (FSF-15), the modulus reduces to 191.39 cN/tex, suggesting that excessive polymer concentrations may affect stiffness owing to structural inconsistencies or reduced filament orientation. This reduction in stiffness is further supported by the breaking elongation data presented in Figure 4c, which shows that the elongation initially decreases from 13% for FSF-5 to 9.2% for FSF-8, stabilizes at ~11% for FSF-10 and FSF-12, and then sharply increases to 18.8% for FSF-15. This inverse relationship between modulus and elongation suggests that while moderate polymer concentrations (FSF-10 and FSF-12) produce the most stiffness and durable FSFs, higher polymer concentrations result in increased ductility and deformation capacity due to the formation of a dense polymer matrix with restricted filament orientation. Figure 4d further supports these findings, as breaking tenacity increases from 4.23 cN/tex for FSF-5 to 21.87 cN/tex for FSF-12, highlighting 12 wt% as the optimal concentration for achieving the maximum mechanical strength. For FSF-15, a slight increase to 22.14 cN/tex and the residual strength after break are shown, indicating that the structural enhancement is limited due to phase separation irregularity at high polymer concentration, and the network structure consisting of strands is relatively more developed. These results indicate that FSF-12 exhibits the most balanced mechanical properties, exhibiting the highest modulus and toughness while maintaining a moderate elongation, whereas FSF-15 exhibits a more ductile fiber structure with reduced stiffness. Thus, it highlights the influence of polymer concentration on the unique network structure of the fabricated FSF and the design optimization of mechanical performance.

**Figure 5:**
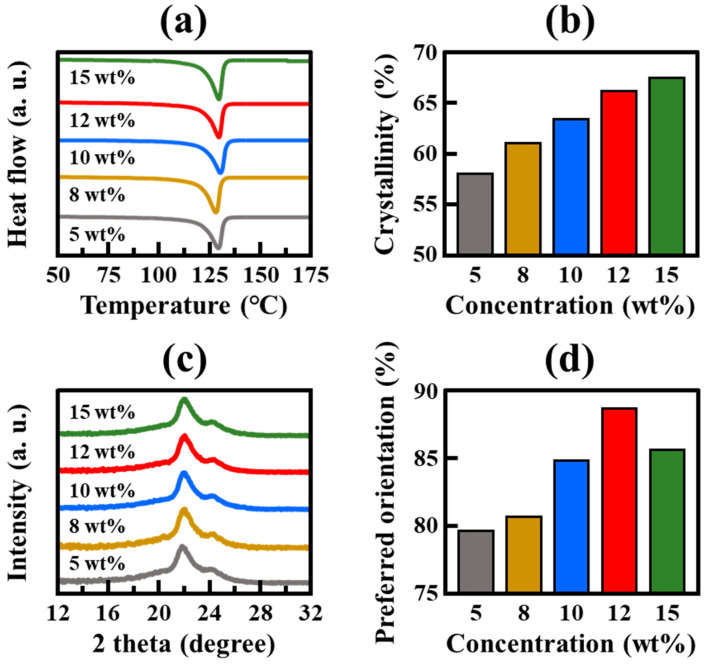
Thermal and crystallographic analysis of FSFs prepared at different HDPE concentrations: (**a**) DSC melting curves; (**b**) crystallinity values derived from DSC; (**c**) XRD patterns; and (**d**) preferred orientations obtained from azimuthal scans at 2θ = 22° using WAXD.

To further investigate the influence of polymer concentration on FSF formation, thermal and crystallographic analyses were conducted (Figure 5), which revealed the mechanism underlying the observed effect of polymer concentration on the phase separation, crystalline domain formation, polymer orientation, and mechanical properties of the FSFs. Figure 5a presents the DSC melting curves, showing that all the FSFs exhibit a T_m_ of approximately 130 °C (characteristic of HDPE), irrespective of the polymer concentration. However, the crystallinity of the FSFs, determined from the melting enthalpy, varies with polymer concentration (Figure 5b) [25,30]. FSF-5 exhibits the lowest crystallinity (57.3%), which increases as the polymer concentration is increased, reaching 65.3% and 66.6% for FSF-12 and FSF-15, respectively. The slight increase in crystallinity from FSF-12 to FSF-15 suggests that although high polymer concentrations provide further nucleation sites, this improvement becomes marginal beyond a certain concentration threshold. This trend indicates that the number of nucleation sites is almost saturated in FSF-12, and further polymer addition results in only limited further crystallization. The WAXD patterns in Figure 5c confirm these trends, displaying two primary diffraction peaks at 21.6° and 23.9°, corresponding to the (110) and (200) crystalline planes of HDPE, respectively [13,31]. FSF-5 exhibits a broad shoulder peak at ~19.3°, which suggests that the initial high proportion of amorphous phase decreases as the polymer concentration increases. This reduction in amorphous content aligns with the increased crystallinity observed in the DSC analysis. FSF-12 and FSF-15 show similar diffraction patterns, indicating that crystallinity maximizes at high polymer concentrations. In addition, the crystal size of FSFs was calculated using the Scherrer’s equation based on the full width at half maximum (FWHM) of the (110) diffraction peak [32,33]. The calculated crystal sizes were as follows: FSF-5 (6.43 nm), FSF-8 (6.91 nm), FSF-10 (6.70 nm), FSF-12 (6.40 nm), and FSF-15 (6.45 nm). These values are notably smaller than the typical HDPE crystal size of 10–20 nm, suggesting that the rapid pressure-induced phase separation (PIPS) limited the time available for crystal growth. This finding supports the notion that the phase separation process rather than thermal equilibrium dominates the microstructural development in FSFs. To assess polymer orientation, WAXD azimuthal scans at 2θ = 22° were performed, and the preferred orientation results are shown in Figure 5d. The preferred orientation increases with concentration, maximizing to 88% for FSF-12 and then slightly decreasing to 85% for FSF-15. This trend correlates with the mechanical property data presented in Figure 4, which shows that tenacity increases with concentration, peaking for FSF-12 before slightly decreasing for FSF-15. Furthermore, when interpreted alongside the DSC results, the crystal size analysis helps to explain the relatively low T_m_ observed in all FSFs. Since T_m_ is closely related to the lamellar thickness or crystal size, the consistently small crystal dimensions (6.4–6.9 nm) across all concentrations account for the lower T_m_ (~130 °C) compared to pristine HDPE (~134 °C) [34,35]. These results indicate that while polymer concentration influences the number of crystalline domains, it does not significantly affect individual crystal size. Consequently, polymer concentration plays a more critical role in regulating the number of nucleation events during phase separation, ultimately shaping the network structure and mechanical performance of FSFs.

**Figure 6:**
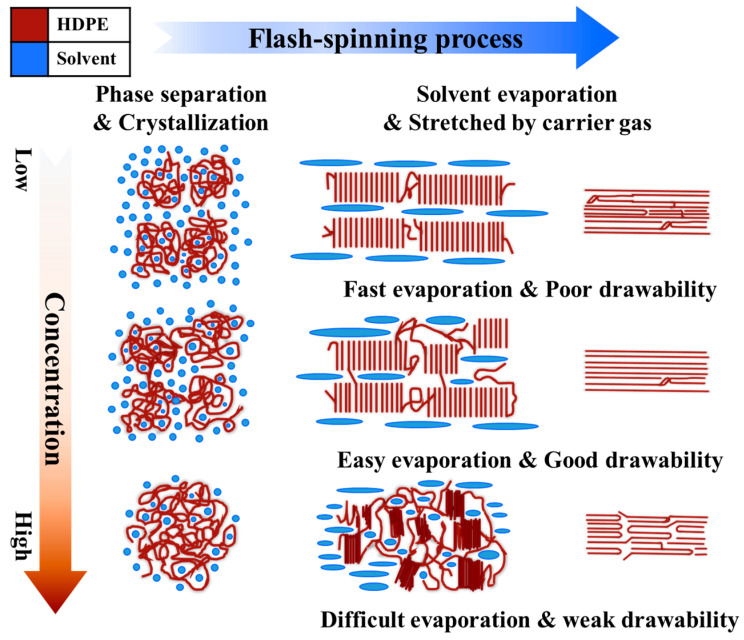
Influence of HDPE concentration and solvent evaporation on crystallization and drawability in the flash-spinning process.

Figure 6 illustrates the mechanism by which polymer concentration influences phase separation, solvent evaporation, and fiber drawability during the flash-spinning process (the left side of Figure 6 illustrates phase separation and crystallization behavior, and the right side depicts solvent evaporation and fiber stretching). At low polymer concentrations, the presence of a large number of solvent–polymer interfaces promotes rapid solvent evaporation, causing fast phase transition that limits polymer chain alignment. This results in poor drawability and low preferred orientation [12,18,26]. In contrast, at moderate polymer concentrations, phase separation produces well-defined crystalline domains, while solvent evaporation occurs at an optimal rate. This balance allows polymer chains to align effectively, leading to better drawability and higher mechanical strength compared to the features observed at low polymer concentrations [36,37]. At high polymer concentrations, the solvent vaporization within the dense polymer network is slowed down, which in turn disrupts the alignment of the polymer chains by slowing down the jet velocity. This reduced alignment results in weaker drawability and lower mechanical performance compared to the features observed at moderate polymer concentrations, despite the increase in crystallinity. These findings highlight the role of the interplay between crystallization and polymer orientation in determining the mechanical properties of FSFs. While FSF-15 exhibits the highest crystallinity, its preferred orientation is lower than that of FSF-12, suggesting that excessive polymer loading disrupts molecular alignment. This misalignment likely contributes to the reduction in modulus and tenacity observed in FSF-15, which shows high crystallinity. The high elongation for FSF-15 suggests that restricted solvent evaporation at high polymer concentrations limits proper fiber alignment, resulting in increased ductility. Consequently, FSF-12 exhibits the most balanced mechanical properties, with optimized crystallinity, polymer orientation, and structural integrity. Further polymer addition beyond this concentration leads to diminishing mechanical performance due to misaligned polymer chains. These results confirm that polymer concentration directly affects phase separation and crystallization during flash spinning, thus affecting the mechanical properties of FSFs. Proper control of polymer concentration is essential for achieving FSFs with optimal fiber strength, stiffness, and flexibility to meet the mechanical requirements of various applications.

## 4. Conclusions

This study demonstrated the critical influence of polymer concentration on the morphology, mechanical properties, and crystallization behavior of flash-spun filaments (FSFs) produced from high-density polyethylene (HDPE). Increasing polymer concentration led to greater filament thickness, enhanced crystallinity, and improved mechanical strength, with the optimal performance observed at 12 wt%, where the modulus peaked at 270.77 cN/tex and elongation was minimized. At this concentration, a well-balanced fiber network with superior strength, stiffness, and structural integrity was achieved. However, further increasing the concentration to 15 wt% resulted in a decline in mechanical properties, including a reduced modulus of 191.39 cN/tex, despite the highest crystallinity of 66.6%. This discrepancy is attributed not to improved crystal growth, but to the difficulty in solvent evaporation within the dense polymer matrix. At high concentrations, limited solvent mobility suppressed jet stream formation and polymer alignment, which in turn compromised filament drawability and molecular orientation. The analysis of crystal size using X-ray diffraction revealed consistently small crystallites (6.4–6.9 nm) across all samples, significantly smaller than typical HDPE crystals (10–20 nm). This indicates that the rapid phase separation induced by a pressure drop during flash spinning limited the time for crystal growth. As a result, polymer concentration primarily influenced the number of nucleation sites, rather than the size of individual crystals, reinforcing the idea that phase separation and evaporation kinetics are central to network structure formation. Overall, these findings highlight the need for precise control of polymer concentration to optimize crystallization behavior, polymer orientation, and mechanical performance in FSFs. A concentration of 12 wt% emerges as a critical threshold for achieving balanced mechanical strength, drawability, and structural cohesion, offering valuable guidance for both academic research and industrial applications of flash-spun nonwovens.

## Figures and Tables

**Table 1:** Linear density of FSFs and diameter and distribution parameters of strands constituting FSFs.

Concentration (wt %)	Fineness (tex)	Average Diameter (μm)	Maximum Diameter (μm)	Minimum Diameter (μm)	Standard Deviation	Variance
5	14.1	4.17	15.5	1.07	2.66	7.07
8	17.7	3.93	16.5	0.99	2.53	6.38
10	21.7	8.36	39.9	1.01	8.16	66.6
12	23.6	10.17	73.5	0.71	11.43	130.6
15	34.4	7.42	39.3	1.64	6.24	38.9

## Data Availability

The original contributions presented in this study are included in the article. Further inquiries can be directed to the corresponding author.
